# Dermatophytosis in a Chilean fox: first case of *Nannizzia gypsea* in *Lycalopex griseus* and the need for a one health approach

**DOI:** 10.1016/j.mmcr.2025.100725

**Published:** 2025-08-13

**Authors:** Ailén Dumont-Viollaz, Leslye Camila del Río, Carolina Sánchez, Pamela Thomson

**Affiliations:** a Escuela de Medicina Veterinaria, Facultad de Ciencias de la Vida, Universidad Andrés Bello, República 440, Santiago, 8370251, Chile; b Programa de Doctorado en Medicina de la Conservación, Universidad Andrés Bello, República 252, Santiago, 8370251, Chile; c Unidad de Rehabilitación de Fauna Silvestre (UFAS), Escuela de Medicina Veterinaria, Universidad Andrés Bello, Av. Lo Pinto s/n, Colina, Santiago, 8370134, Chile; d One Health Institute, Faculty of Life Sciences, Universidad Andres Bello, Santiago, 8370251, Chile

**Keywords:** Chilla fox, Mycoses, Dermatophytosis, Antifungal susceptibility

## Abstract

Fungal diseases, despite their impact on animal and human health, are often underestimated. *Nannizzia gypsea*, a geophilic dermatophyte, has been detected in humans, dogs, and cats, as well as in some wild animals. We present the first clinical case of superficial mycosis caused by *N. gypsea* in a wild fox (*Lycalopex griseus*). Effective treatment consisted of a topical ketoconazole. Despite previous reports of resistance to antifungals, the strain showed susceptibility to all those tested. Given the characteristics of the causative agent and the environment where the fox was found, we emphasize the need to contain these events from a public health perspective.

## 1 Introduction

The South American grey fox or chilla fox, *Lycalopex griseus* (Grey, 1837), is one of three native fox species found in Chile. Nationally, it ranges from the Arica and Parinacota Region to the Magallanes and Chilean Antarctic Regions, and inhabits steppes, grasslands, and scrublands. It is classified as Least Concern both internationally by the IUCN and nationally by the Ministry of the Environment of Chile [1,2], however, there is a need to deepen our knowledge of various aspects of these animals, particularly regarding their diseases. Recent studies have reported on viruses, bacteria, and parasites affecting *L. griseus* this country [3,4]. Furthermore, although mycoses have been reported in foxes throughout the world, there are no reports of fungal infections in *L. griseus* to date.

Dermatophytes, of which 59 species have been described so far, are a group of keratinolytic and keratinophilic fungi and the cause of most superficial mycoses. They are filamentous, septate, and hyaline fungi. They possess keratinases that allow them to use keratin as a source of nitrogen, thus primarily affecting the skin, hair, and nails of animals and humans [5]. Clinical presentations vary between hosts. In humans, alopecic or inflamed focal lesions are observed. Canine dermatophytosis is usually characterized by focal alopecic lesions and brittle hairs with seborrhea, sometimes dry, focal, or multifocal crusting dermatitis, kerion, and onychomycosis [6]. In canine dermatophytosis, the most frequently isolated etiological agents are *Microsporum canis* followed by *Nannizzia gypsea* [7].

*N. gypsea* (Nannizzi, 1927) (formerly *Microsporum gypseum*) is one of the 13 species that make up the genus. It is a geophilic, cosmopolitan species that normally lives in the soil. It can cause dermatophytosis in humans and animals, the latter also having zoonotic potential with respect to the fungus [8,9]. It is characterized by fast-growing, powdery colonies, cinnamon-colored with a yellowish reverse, sometimes with pink tints. Regarding its micromorphology, it presents macroconidia in large clusters with thin walls, regularly warty, 3–6 (−8) cells, fusiform, 25–60 × 8.5–15.0 μm. Microconidia are sessile or pedunculated, with smooth, thin walls, clavate, and measuring 3.5–8.0 × 2–3 μm [8]. In addition, the use of molecular techniques that allow for reliable species identification is suggested [9]. To date, no mycoses caused by *N. gypsea* have been reported in foxes. This work reports the first case of mycosis in *L. griseus*, and the first case of *N. gypsea* in foxes.

## 2 Case presentation

In November 2024, a 2-year-old male chilla fox (*L. griseus*) was admitted to the Wildlife Unit (UFAS) of the Andrés Bello University (Chile), rescued by the Agricultural and Livestock Service (SAG) from a private house, located on the outskirts of the Metropolitan Region (Central Chile, 33°41′08″S 71°12′53″W).

**Fig. 1:**
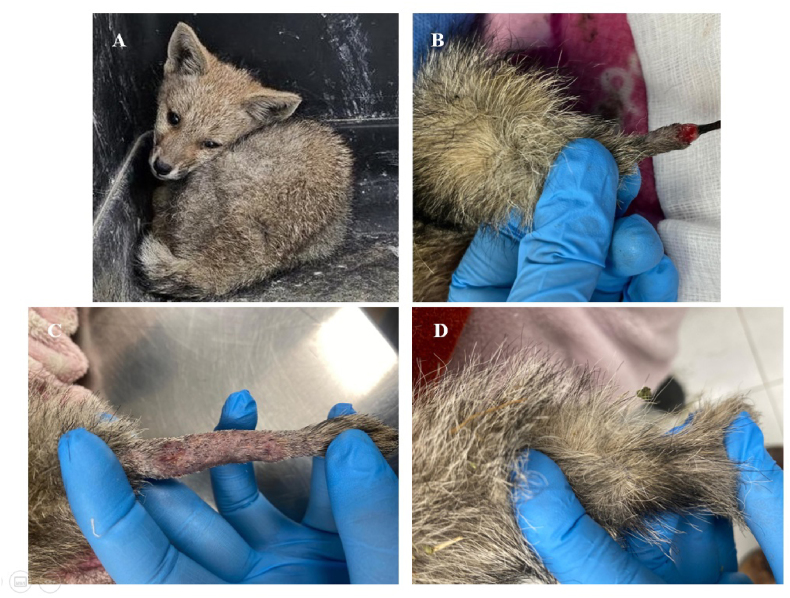
A: Chilla fox (*L. griseus*) on the day of admission to the wildlife unit. B: Necrotic and crusted lesion on the distal end of the tail. C: Alopecia, slightly rounded and scaly lesions (day +28) on the tail D: Appearance of the tail at the end of treatment with topical ketoconazole (day +79).

On admission to the unit (day 0), the fox had a body condition score of 2/5, a shaggy coat, 9 % dehydration, pale mucous membranes, and moderate ocular discharge (Fig. 1A). Clinical examination revealed a single crusted lesion associated with tissue necrosis on the distal part of the tail extending 1.8 cm (Fig. 1B). Antigen detection for distemper virus and parvovirus (Virbac, Santiago, Chile) was performed, with negative results. The fox was rehydrated and fed. The wound was managed by surgical cleaning, removing necrotic tissue until granulation tissue was visible. Cleansing with saline solution and washing solution (Actimaris, Izasa Medical, Barcelona, Spain) was indicated once daily for 7 days. At the evaluation on day +8, it was indicated to apply paraffin gauze every 48 hours for 14 days (Reuter, Santiago, Chile). At the end of this period (day +22), the fox remained under observation within the enclosure, with permanent clinical evaluations.

On day +28, scar tissue was observed, as well as new, rounded, alopecic, and slightly scaly lesions on the tail (Fig. 1C). A sample of hair and scales was taken from the lesions for mycological analysis. While awaiting results, staff were instructed to wash the fox's tail with miconazole shampoo (Regepipel plus, Drag Pharma, Santiago, Chile) twice a week.

**Fig. 2:**
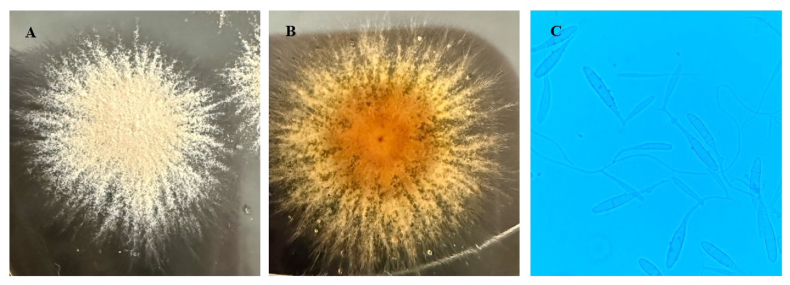
A and B: Surface and reverse of *Nannizzia* sp. grown on Sabouraud dextrose agar. A powdery expanding colony is observed, whitish tan in color with a yellowish reverse. C: Abundant macroconidia, thin-walled, spindle-shaped, regularly warty, with 3–8 septa per cell.

Analysis of the sample was carried out at the Clinical Microbiology and Microbiome Laboratory of Andrés Bello University. A direct microscopic examination of the hair and scales was conducted using 10 % lactophenol. The sample was also plated on Sabouraud dextrose agar with chloramphenicol and cycloheximide and incubated at 25 °C for 15 days. The macro- and microscopic characteristics of the colony allowed for the identification of *Nannizzia* sp (see Fig. 2).

The DNA extraction from a new colony was performed with the Quick-DNA Fungal/Bacterial Miniprep Kit (ZYMO Research, Irvine, CA, USA), and the DNA was quantified using a Nanodrop 2000c nanospectrophotometer (ThermoScientific, Waltham, MA, USA). Subsequently, 10 ng of the extracted DNA were used for PCR amplification of the nuclear ribosomal internal transcribed spacer (ITS) region, using the primers ITS4 5′-TCCTCCGCTTATTGATATGC-3′ and ITS5 5′-GGAAGTAAAAGTCGTAACAAGG-3′ and, for the calmodulin gene, using the primers Cmd5 5′-CCGAGTACAAGGARGCCTTC-3′ and Cmd6 5′-CCGATRGAGGTCATRACGTGG-3′ [10]. The sequences obtained from the PCR-amplified regions were compared with freely available sequences from GenBank using the basic local alignment search tool (BLAST). Confirmation of the correct identification of the pathogenic species was based on a percentage of identity and coverage >99 %, validating the presence of *N. gypsea*, whose GenBank accession codes are PV558824 and PV555436 for ITS and calmodulin, respectively. In parallel, the antifungal susceptibility was determined with the diffusion method according to the M38-A2 guidelines [11] using Ketoconazole (15 μg - Rosco Diagnostica A/S, Taastrup, Denmark) Amphotericin B (10 μg - Liofilchem, Italy), Fluconazole (25 μg - Rosco Diagnostica A/S, Taastrup, Denmark), Clotrimazole (10 μg - Liofilchem, Italy), Miconazole (10 μg - Liofilchem, Italy), Itraconazole (50 μg - Liofilchem, Italy) and Voriconazole (1 μg - Liofilchem, Italy), showing susceptibility to all of them.

Consequently, on day +49, the patient was started on topical 2 % ketoconazole cream (Behvazan, Tehran, Iran), administered twice daily for 4 weeks by the center's technical staff. At the end of the period (day +79), complete remission of the lesions was observed (Fig. 1D), and mycological cure was achieved 15 days later. The fox has not shown any recurrence five months after the end of treatment. The fox currently resides at UFAS until its release into the wild. Its enclosure measures 3 m high, 3 m wide, and 6 m long; it is made of metal and concrete, covered with earth, with shelters, logs, and raised wooden platforms. The cleaning and disinfection protocol is performed weekly and consists of removing biological waste, using detergent to eliminate organic matter, and applying quaternary ammonium dilution 1:50 (Detsaval, Avalco, Santiago, Chile).

## 3 Discussion

We report the first case of mycosis in *L. griseus* and the first case of dermatophytosis caused by *N. gypsea* in foxes.

*Nannizzia gypsea*, one of the species that causes dermatophytosis, is a geophilic fungus with a worldwide distribution. It has been described as an infectious agent in humans and domestic animals such as dogs, cats, sheep, and horses. However, reports in wild animals are associated only with hedgehogs [12,13].

Dogs, cats, and hedgehogs have also been shown to act as dermatophyte reservoirs and can effectively disseminate the fungus to other species or the environment [1,[14], [15], [16]]. Likewise, the increasingly close contact between domestic, wild, and human animals increases the likelihood of transmission of infectious agents, including these fungi [13]. This situation is promoted by human activities such as urbanization, land-use change, and deforestation [17]. This is consistent with what is happening in central Chile, where the dog and cat populations are increasing, and the number of stray dog sightings are high [18]. The authors do not rule out the possibility that the aforementioned factors are directly or indirectly related to the report of this case.

Regarding the susceptibility analysis, the *N. gypsea* isolate showed susceptibility to all the antifungals evaluated, which has positive implications for the choice of treatment and the possibility of modifying it if necessary. Despite this, increasingly frequent antifungal resistance is a reality we face, and one that is not uncommon in dermatophytes such as *N. gypsea* and other dermatophyte species [19]. In this regard, new therapeutic options, and a thorough study of host-pathogen interactions in relation to the human impact on them are needed.

Future research is expected to deepen our understanding of the dynamics between fungal pathogens, such as *N. gypsea*, and their interactions with domestic, wild, and human animals [20]. For now, the authors emphasize the importance of reporting cases associated with these types of agents, considering the “One Health” approach.

## CRediT authorship contribution statement

**Ailén Dumont-Viollaz:** Writing – review & editing, Writing – original draft, Methodology, Formal analysis. **Leslye Camila del Río:** Writing – original draft, Methodology, Investigation. **Carolina Sánchez:** Writing – original draft, Project administration, Methodology. **Pamela Thomson:** Writing – review & editing, Methodology, Investigation, Funding acquisition, Formal analysis, Conceptualization.

## Consent

Not applicable.

## Sources of funding

This work received financial funding from the Agencia Nacional de Investigación y Desarrollo (10.13039/501100020884ANID, Chile) by grant Iniciacion 10.13039/501100002850FONDECYT 11231174.

## Declaration of competing interest

There are none.
